# Dual-Source CT Angiography of Peripheral Arterial Stents: In Vitro Evaluation of 22 Different Stent Types

**DOI:** 10.1155/2011/103873

**Published:** 2011-07-18

**Authors:** Michael Köhler, Matthias C. Burg, Alexander C. Bunck, Walter Heindel, Harald Seifarth, David Maintz

**Affiliations:** Department of Clinical Radiology, University of Münster, Albert-Schweitzer-Stra*β*e 33, 48129 Münster, Germany

## Abstract

*Purpose*. To test different peripheral arterial stents using four image reconstruction approaches with respect to lumen visualization, lumen attenuation and image noise in dual-source multidetector row CT (DSCT) in vitro. *Methods and Materials*. 22 stents (nitinol, steel, cobalt-alloy, tantalum, platinum alloy) were examined in a vessel phantom. All stents were imaged in axial orientation with standard parameters. Image reconstructions were obtained with four different convolution kernels. To evaluate visualization characteristics of the stent, the lumen diameter, intraluminal density and noise were measured. *Results*. The mean percentage of the visible stent lumen diameter from the nominal stent diameter was 74.5% ± 5.7 for the medium-sharp kernel, 72.8% ± 6.4 for the medium, 70.8% ± 6.4 for the medium-smooth and 67.6% ± 6.6 for the smooth kernel. Mean values of lumen attenuation were 299.7HU ± 127 (medium-sharp), 273.9HU ± 68 (medium), 270.7HU ± 53 (medium-smooth) and 265.8HU ± 43. Mean image noise was: 54.6 ± 6.3, 20.5 ± 1.7, 16.3 ± 1.7, 14.0 ± 2 respectively. *Conclusion*. Visible stent lumen diameter varies depending on stent type and scan parameters. Lumen diameter visibility increases with the sharpness of the reconstruction kernel. Smoother kernels provide more realistic density measurements inside the stent lumen and less image noise.

## 1. Introduction

Arterial stenoses and occlusions in different regions of the body are frequently treated with angioplasty and stent implantation. After stent implantation, there is a risk of in-stent restenosis which can be caused by neointimal proliferation, vessel wall inflammation, or stent thrombosis [1, 2]. Therefore follow-up examinations are needed after successful revascularization for detecting restenosis. 

At present, there are different imaging techniques available. Digital subtraction angiography (DSA) has been the standard modality for evaluating stent patency for a long time. However, its invasiveness is afflicted with possible complications and a less invasive alternative is eligible. Duplex sonography of arteries is noninvasive but highly operator dependant. 

Three-dimensional contrast-enhanced MR angiography (MRA) is a frequently used noninvasive alternative to DSA for screening patients [3, 4]. However, MRA of stented arteries is difficult because stent-related susceptibility artifacts may disturb stent lumen visibility [5–7]. Hamer et al. concluded in their studies in 2005 and 2006 that MRA is not yet a reliable technique to characterize in-stent stenoses [8, 9]. 

Spiral computed tomography angiography (CTA) is another noninvasive method to evaluate peripheral arteries and has been shown to be an alternative to intra-arterial DSA in a postinterventional followup [10]. It was shown that CTA provides comparable findings to intra-arterial DSA for the detection of stenoses in renal arteries [11]. Studies with 16-row CT scanners showed promising results regarding assessability of in-stent stenosis with CTA [12–14].

Modern CT scanners are equipped with 64 or more detector rows and increased gantry rotation speed to allow the acquisition of more slices with a better spatial resolution in a shorter time. Clinical data regarding stent evaluation are available as single center data as well as a meta-analysis [14, 15]. The purpose of this study was to investigate the stent lumen visibility and artifacts of different peripheral stents for different locations of the body in a CT angiography examination. Furthermore, the study aimed at comparing different CT reconstruction kernels with regard to stent imaging.

## 2. Material and Methods

### 2.1. Evaluated Stents and Experimental Setup

Twenty-one different stents and one stentgraft of different material and design were studied. Manufacturer, material, design, length, and nominal diameter of the stents and stent graft are summarized in Table 1. Ten stents were made of Nitinol, seven of stainless surgical steel (316 L), two of a cobalt-based alloy, two of tantalum, and one of a platinum alloy. The Wallstent was made of a cobalt-based alloy covered by polyethylene (PET). 

The stents and the stentgraft were inserted in plastic tubes with 5, 7, 8, 10, or 13 mm lumen diameter exactly matching their nominal diameter with one exception: the Palmaz Genesis Stent had a nominal diameter of 6 mm and was implanted into a 5 mm plastic tube. The wall of the small tubes (5, 7, and 8 mm) had a thickness of <0.3 mm, and the material of the bigger tubes (10 and 13 mm) was about 1 mm thick.

The tubes were filled with contrast material (Ultravist 300, Schering AG, Berlin, Germany) diluted to 250 HU, closed at both ends and positioned in a plastic container filled with vegetable oil. The density of the oil was adjusted to −70 HU by addition of Lipiodol Ultrafluid (Byk Gulden, Konstanz, Germany) to simulate perivascular fat. The tubes with implanted stents and stentgraft were then positioned in the gantry in an orientation parallel to the *z*-axis of the scanner.

### 2.2. Dual-Source CT Parameters

DSCT images were acquired on a current dual-source system (Somatom Definition, Siemens Medical Solutions, Forchheim, Germany) with a detector collimation of 2 × 32 × 0.6 mm and fixed pitch of 14.4 mm/sec. Rotation time was 330 msec, effective tube current 120 mAs, and tube voltage 120 kV. Four different image reconstructions were obtained using a fixed field-of-view of 170 mm and matrix of 512 × 512: (1) a smooth kernel (B20f), (2) a medium-smooth kernel (B30f), (3) a medium kernel (B40f), and (4) a medium-sharp kernel (B50f).

Axial images with a slice thickness of 0.6 mm were used for the evaluation. Secondary multiplanar reformations (MPRs) were created for demonstration purposes only.

### 2.3. Evaluation of Visualization Characteristics of the Stent: Lumen Diameter, Intraluminal Density, and Noise

**Figure 1:**
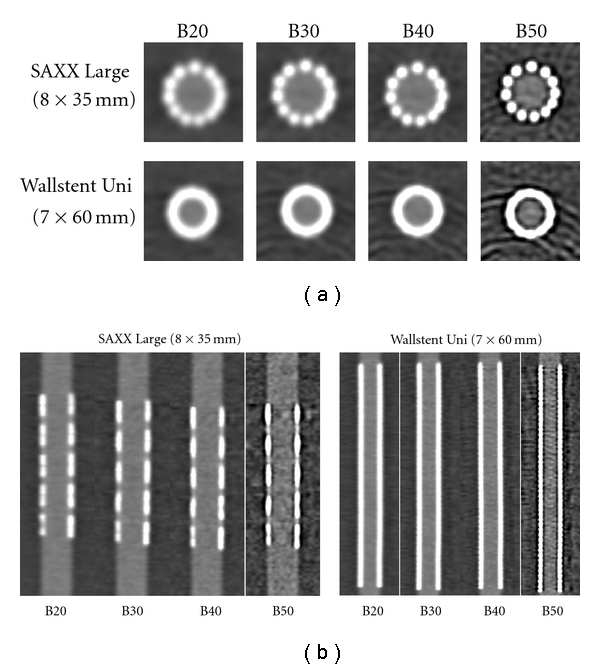
Comparison of the four different reconstruction protocols. Exemplary axial (a) and through-plane (b) reformations of the SAXX Large and the Wallstent Uni in the four applied reconstruction protocols B20f, B30f, B40f, and B50f. Note the increase of the visible lumen diameter and the increase of noise using the B50f reconstruction kernel.

**Figure 2:**
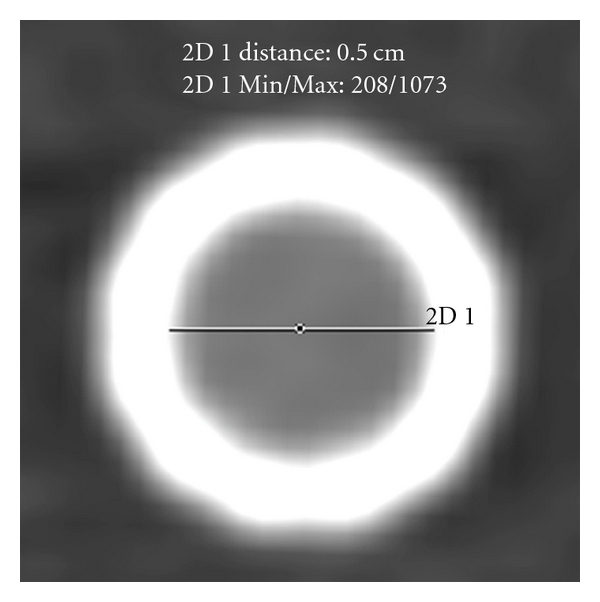
Positioning of the electronic caliper for measuring the visible stent lumen (Wallstent Uni, B40f, result: 5.0 mm).

Axial reformations of all stents were evaluated in a window width of 1500 HU and a center of 300 HU as shown for the SAXX Large and the Wallstent Uni in Figure 1. This setting has proven to be useful for the evaluation of coronary stents in previous studies [16, 17]. The diameter of the visible stent lumen in the center of the stent and on two adjacent images was measured as shown in Figure 2 using the electronic caliper for distance measurements provided with the CT system's standard software. From these three measurements a mean value for each stent was calculated. Attenuation values inside the visible stent lumen were measured by a region of interest technique (ROI) in the same three images to calculate a mean stent lumen attenuation. The ROI with a size of 12 pixels was placed in the center of the visible stent lumen without inclusion of the stent struts or streak artifacts.

Image noise was defined as the standard deviation of a ROI density measurement outside the vessels in the surrounding oily fluid. 

All measurement results are displayed as mean, standard deviation, and range. As the measurement results for visible lumen diameter, lumen attenuation, and image noise proved not to be normally distributed within the groups of the four different kernels the nonparametric Friedman test was used to check for overall differences among the reconstruction methods. 

A posthoc analysis was carried out with a paired comparison between all kernels using the Wilcoxon test. At *P* < .05 statistical significance was assumed.

**Figure 3:**
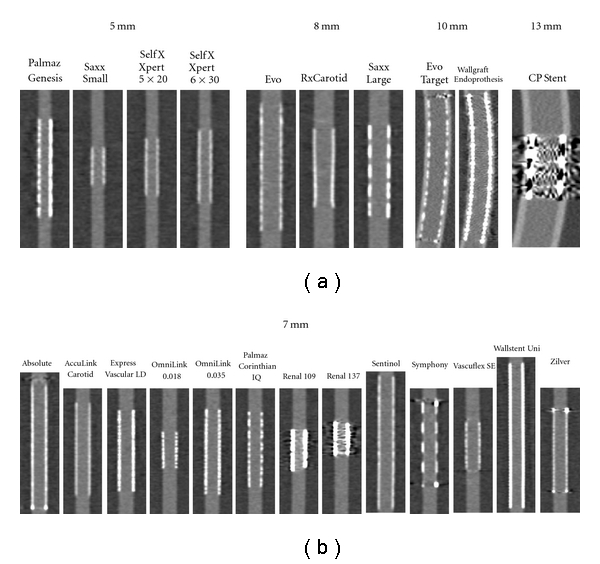
Comparison of 22 different peripheral arterial stents. Longitudinal through-plane reformations of 0.6 mm slice thickness reconstructed using a B40f medium kernel are shown.

Secondary multiplanar reformations (MPRs) were created for demonstration purposes only (Figure 3).

## 3. Results

### 3.1. Diameters of the Visible Stent Lumen

The visible stent diameters using the four reconstruction protocols are summarized in Table 2.

Using the B20f kernel reconstruction, the visible lumen diameter ranged from 49.0% in the Renal 137 stent to 77.3% in the Evo Target stent, and the mean visible lumen diameter was 67.6 ± 6.6%.

Using the B30f kernel for image reconstruction, the visible lumen diameter ranged from 52.4% in the Renal 137 stent to 81.7% in the Evo Target stent (mean 70.8 ± 6.4%).

Using the B40f kernel, the visible lumen diameter ranged from 53.3% in the Renal 137 stent to 83.7% in the Evo Target stent (mean 72.8 ± 6.4%).

With the B50f kernel the visible lumen diameter ranged from 57.1% in the Renal 137 stent to 83.3% in the Evo Target stent (mean 74.5 ± 5.7%). In this reconstruction severe artifacts were found especially within the stents Renal 109, Renal 137, and CP Stent, so that the stent lumen could not be evaluated.

In this protocol the Evo Target (83.3%), the CP Stent (81.3%), the Evo (80.4%), and the Sentinol (80%) showed the best lumen visibility of ≥80% of the stent lumen, but, as stated before, the CP stent showed severe artifacts within the depicted stent lumen. 16 stents showed a good lumen visibility of 69–77%. Only the two tantalum stents Renal 109 (63.9%) and Renal 137 (57.1%) showed a lumen visibility of less than 66.6%.

Differences between all the kernels were highly significant with *P* < .01 in the Wilcoxon test. The improvement of the stent lumen visibility by using sharper kernel reconstructions can be estimated in Figure 1.

### 3.2. Attenuation of the Stent Lumen

The attenuation values of the stent lumen using the different reconstruction protocols are summarized in Table 2. The Friedman test indicated no significant differences among the reconstruction kernels for the parameter lumen attenuation, but it showed significant differences among the reconstruction kernels for the parameters visible lumen diameter and noise. The B20f kernel reconstruction resulted in a lumen density ranging from 222.2 HU in the CP Stent to 432.6 HU in the Renal 109 Stent (mean 265.8 ± 43.1 HU).

In the B30f kernel reconstruction the lumen density ranged from 216.9 HU in the Express Vascular LD to 436.9 HU in the Renal 109 Stent (mean 270.1 ± 53.2 HU).

Using the B40f kernel reconstruction, the lumen density ranged from 216.6 HU in the SAXX Large to 457.4 HU in the CP Stent (mean 273.9 ± 68.4 HU).

The B50f kernel reconstruction resulted in a lumen density range from 176.2 HU in the SAXX Small to 643.3 HU in the Renal 137 Stent (mean 299.7 ± 127.2 HU). 

The most realistic measurement of the mean stent lumen attenuation (closest to the actual 250 HU) was achieved with the B20f smooth kernel reconstruction protocol, but the improvement compared to all other reconstruction protocols was not significant in the Friedman test or in the paired Wilcoxon test (*P* > .15 for all tests).

In the B20f reconstruction kernel 18 stents showed a deviation of the attenuation values of less than 10% (density between 225 and 275 HU) from the expected attenuation of 250 HU: Saxx Small, SelfX Xpert, Absolute, AccuLink Carotid, Express Vascular LD, OmniLink 0.018, OmniLink 0.035, Palmaz Corinthian IQ, Sentinol, Symphony, Vascuflex SE, Wallstent Uni, Zilver, Evo, RxCarotid, Saxx Large, Evo Target, and Wallgraft Endoprothesis. Only four stents showed a bigger deviation of the attenuation values: Palmaz Genesis (12%), Renal 109 (73%), Renal 137 (31%), and CP Stent (11%).

### 3.3. Noise

Mean noise values of the four reconstruction kernels are summarized in Table 2. The noise increased from the smooth to the sharper kernels: 14.0 ± 2.0 HU in B20f, 16.3 ± 1.7 HU in B30f, 20.5 ± 1.7 HU in B40f, and 54.6 ± 6.3 HU in B50f. The differences between the examined reconstruction protocols were highly significant with *P* < .01 in the Friedman test and all the paired Wilcoxon tests.

### 3.4. Comparison of Lumen Visibility and Lumen Attenuation between the Different Materials

We compared the results of the stents depending on their material. The results are shown in Table 3. We found very similar values for the materials 316 L and Nitinol. In the B40f reconstruction both showed a lumen visibility of 74.4% and realistic attenuation values of 249.4 HU (316 L) and 242.4 HU (Nitinol), respectively. The Cobalt alloy exhibited only slight differences with a lumen visibility of 73.2% and a lumen attenuation of 273.9 HU with the B40f reconstruction. The platinum-iridium stent showed a lumen visibility of 77.7% and a lumen attenuation of 457.4 HU in B40f reconstruction. The tantalum stents showed a lumen visibility of 56.4% and a lumen attenuation of 425.6 HU in the B40f reconstruction protocol.

## 4. Discussion

In the present study a considerable number of different peripheral stents were examined in a state-of-the-art CT system regarding their lumen visibility using CT angiography (CTA). The investigated stents were made from different materials (stainless steel (316 L), Nitinol, Cobalt alloy, Tantal, and Platinum-Iridium alloy) and for different areas of applications (arterial and peripheral arteries, in general, biliary duct, carotid arteries, renal arteries, iliac arteries, femoral arteries and aorta). Our study shows that CTA can be used for follow-up examinations in the majority of the evaluated stents. However, the lumen visibility differs extensively between different stent types. 

The phantom used was designed to simulate conditions comparable to an in vivo CTA examination. Nevertheless, some limitations have to be considered. In all scans the stents were positioned parallel to the *z*-axis of the scanner. This would resemble the in vivo position of an aortic stent but not that of renal or iliac stents. Several groups have shown that stent artifacts of coronary stents depend on the angle between stent and scanner [18, 19]; therefore the visible stent lumen in an in vivo study may differ from our results. 

We used a static fluid model, but the effect of flow on the artifact expression should be negligible because CTA works with differences in radiopacity and not with flow parameters. 

In one stent the nominal diameter did not exactly match the diameter of the “vessel”: the 6 mm Palmaz stent was implanted in a 5 mm tube. This was necessary because no appropriate 6 mm tube was available. The possible effect of stent strut compaction when implanting a large diameter stent in a smaller diameter lumen should be minor in this case but must be considered.

It was stated before that the artifacts depend on the stent design and material. Our study demonstrates that stents made from stainless steel (316 L) or Nitinol show the best results in CTA compared to other stents we examined. The Cobalt alloy stents also showed good results in our study. The Platinum-Iridium alloy stent and the tantalum stents showed poorer results, because “blooming” artifacts obscured parts of the stent lumen. The higher magnitude of artifacts in these stents may be mainly due to the higher atomic number of platinum (78) and tantalum (73) when compared to steel (26), cobalt (27), chromium (24), or nickel (28). Therefore, it is useful to know what kind of stent was implanted before the CT examination. 

Our study showed better results for lumen visibility with a modern state-of-the-art CT scanner with 64 detectors than previous studies with a four-slice scanner [20, 21]. Eichhorn et al. stated that image quality rises with number of detectors and the diameter of the stent [22]. Our study underlines that sharper kernels show better lumen visibility than smoother ones as Heuschmid et al. [23] stated. Furthermore in our study the B40f reconstruction showed the best compromise between lumen visibility and noise. The increase of lumen visibility of B50f compared to B40f is only 2.3%. 

The effect of high pitch protocols on coronary artery stent imaging has been investigated [24]; results for iliac artery stents remain to be published. A recent study shows the feasibility of a low-dose protocol for detecting in-stent restenoses of iliac artery stents [25]. 

In summary, all investigated stents seem to be suitable for the evaluation of high-grade stenoses (lumen visibility >50%). Except for the tantalum stents it should even be possible to detect smaller stenoses (lumen visibility >66%). After all CTA with a modern CT scanner seems to be a helpful noninvasive method for the follow-up examination of stented arterial stenoses.

## Figures and Tables

**Table 1:** Name, manufacturer, material, primary area of use, diameter, and length of the examined stents.

No.	Name	Manufacturer	Material	Primary area of use	Diameter (mm)	Length (mm)
1	SAXX Small	Devon	316 L	Arterial vessels	5	17
2	SelfX Xpert	Abbott	Nitinol	Peripheral vasculature/ biliary duct	5	20
3	Palmaz Genesis	Cordis	316 L	Peripheral arteries below aortic arch/biliary tree	6	39
4	Absolute	Guidant	Nitinol	Biliary/peripheral vessels	7	60
5	AccuLink Carotid	Guidant	Nitinol	Internal carotid and common carotid arteries	7	40
6	Express Vascular LD	Boston Scientific	316 L	Peripheral vessels	7	37
7	OmniLink 0,018	Guidant	316 L	Biliary/peripheral arteries	7	18
8	OmniLink 0,035	Guidant	316 L	Biliary/peripheral arteries	7	35
9	Palmaz Corinthian IQ	Cordis	316 L	Peripheral vessels	7	40
10	Renal109	Abbott	Tantal	Renal artery	7	18
11	Renal137	Abbott	Tantal	Renal artery	7	18
12	Sentinol	Boston Scientific	Nitinol	Peripheral vessels	7	59
13	Symphony	Boston Scientific	Nitinol	Iliac artery	7	40
14	Vascuflex SE	B. Braun	Nitinol	Peripheral vessels	7	20
15	Wallstent Uni	Boston Scientific	Cobalt-Superalloy	Iiliac artery, SFA (Superficial Femoral Artery), Tracheal	7	60
16	Zilver	Cook	Nitinol	Carotid artery	7	40
17	Evo	pfm	Nitinol	Pelvic arteries and peripheral vessels/biliary	8	50
18	RxCarotid	Abbott	Nitinol	Carotid artery	8	30
19	SAXX Large	Devon	316 L	Arterial vessels	8	35
20	Evo Target	pfm	Nitinol	Intravascular/biliary	10	80
21	Wallgraft Endoprothesis	Boston Scientific	braided polyester graft bonded to the outside of a Wallstent (Cobalt-Superalloy)	Trachea/bronchus (off label: peripheral arteries)	10	70
22	CP Stent	pfm	90% platinum 10% iridium	Aorta	13	28

**Table 2:** Mean visible lumen diameters and intraluminal attenuation of the investigated stents using four different reconstruction kernels. Diameters of the visible stent lumen are given as mean (minimum/maximum) ± standard deviation in % of the actual lumen. Attenuation and noise values are given as mean (minimum/maximum) ± standard deviation in Hounsfield units (HUs).

Stent name	B20f		B30f		B40f		B50f	
	Diameter (%)	Density (HU)	Diameter (%)	Density (HU)	Diameter (%)	Density (HU)	Diameter (%)	Density (HU)
SAXX Small	66,6	262,9	69,4	263,8	71,4	236,4	74,6	176,2
SelfX Xpert	67,4	263,1	71,4	257,3	73,4	252,7	74,6	270,9
Palmaz Genesis	65,4	279,9	69,4	273,7	70,0	281,2	68,6	194,4
Absolute	65,3	234,5	67,1	233,1	71,9	229,8	74,7	269,4
AccuLink Carotid	60,4	260,0	65,7	278,3	67,6	258,3	71,9	254,6
Express Vascular LD	71,4	244,4	73,9	216,9	76,7	243,4	77,1	230,6
OmniLink 0,018	67,6	265,6	71,9	252,6	75,7	251,0	76,1	253,0
OmniLink 0,035	69,6	249,7	72,9	232,9	76,1	264,3	76,1	235,5
Palmaz Corinthian IQ	71,4	265,7	73,3	255,7	74,3	252,8	74,3	244,1
Renal109	52,4	432,6	56,7	436,9	59,6	436,5	63,9	586,8
Renal137	49,0	327,1	52,4	345,9	53,3	414,6	57,1	643,3
Sentinol	70,4	234,5	76,1	233,5	76,7	239,1	80,0	212,2
Symphony	68,1	254,2	71,9	288,6	73,3	243,8	75,3	212,2
Vascuflex SE	68,1	244,6	72,4	219,5	75,3	234,7	76,7	245,1
Wallstent Uni	68,1	273,5	69,6	269,1	71,0	262,7	71,0	241,1
Zilver	69,0	263,7	71,4	234,7	74,7	219,9	76,7	226,3
Evo	76,3	256,7	76,6	256,8	77,9	247,5	80,4	284,9
RxCarotid	66,3	268,4	68,4	250,3	69,1	257,9	72,1	293,4
SAXX Large	72,9	243,9	76,6	240,6	76,6	216,6	76,6	272,2
Evo Target	77,3	228,3	81,7	245,8	83,7	239,8	83,3	311,8
Wallgraft Endoprothesis	72,3	271,6	73,0	276,5	75,3	285,1	76,0	396,8
CP Stent	72,3	222,2	76,4	379,3	77,7	457,4	81,3	538,7

Mean	**67,6**	**265,8**	**70,8**	**270,1**	**72,8**	**273,9**	**74,5**	**299,7**
Min	**49,0**	**222,2**	**52,4**	**216,9**	**53,3**	**216,6**	**57,1**	**176,2**
Max	**77,3**	**432,6**	**81,7**	**436,9**	**83,7**	**457,4**	**83,3**	**643,3**
SD	**6,6**	**43,1**	**6,4**	**53,2**	**6,4**	**68,4**	**5,7**	**127,2**

**Table 3:** Mean visible lumen diameters and lumen attenuation in the different reconstruction protocols depending on the stent material.

		B20f		B30f		B40f		B50f	
	*n*	Density (HU)	Diameter (%)	Density (HU)	Diameter (%)	Density (HU)	Diameter (%)	Density (HU)	Diameter (%)
316 L	7	258,9	69,3	248,0	72,5	249,4	74,4	229,4	74,8
Nitinol	10	250,8	68,9	249,8	72,3	242,4	74,4	258,1	76,6
Cobalt Superalloy	2	272,6	70,2	272,8	71,3	273,9	73,2	319,0	73,5
Tantal	2	379,9	50,7	391,4	54,6	425,6	56,4	615,1	60,5
Platinum-Iridium Alloy	1	222,2	72,3	379,3	76,4	457,4	77,7	538,7	81,3
